# Associations of Erythrocyte Fatty Acids in the *De Novo* Lipogenesis Pathway with Proxies of Liver Fat Accumulation in the EPIC-Potsdam Study

**DOI:** 10.1371/journal.pone.0127368

**Published:** 2015-05-18

**Authors:** Simone Jacobs, Susanne Jäger, Eugene Jansen, Andreas Peter, Norbert Stefan, Heiner Boeing, Matthias B. Schulze, Janine Kröger

**Affiliations:** 1 Department of Molecular Epidemiology, German Institute of Human Nutrition Potsdam-Rehbrücke, Nuthetal, Germany; 2 German Center for Diabetes Research (DZD), Germany; 3 Center for Health Protection, National Institute for Public Health and the Environment, Bilthoven, Netherlands; 4 Department of Internal Medicine, Division of Endocrinology, Diabetology, Nephrology, Vascular Disease, and Clinical Chemistry, University Hospital of the Eberhard Karls University, Tübingen, Germany; 5 Institute for Diabetes Research and Metabolic Diseases of the Helmholtz Centre Munich at the University of Tübingen (IDM), Tübingen, Germany; 6 Department of Epidemiology, German Institute of Human Nutrition Potsdam-Rehbrücke, Nuthetal, Germany; INRA, FRANCE

## Abstract

**Background:**

Biomarker fatty acids (FAs) reflecting *de novo* lipogenesis (DNL) are strongly linked to the risk of cardiometabolic diseases. Liver fat accumulation could mediate this relation. There is very limited data from human population-based studies that have examined this relation.

**Objective:**

The aim of this study was to investigate the relation between specific FAs in the DNL pathway and liver fat accumulation in a large population-based study.

**Methods:**

We conducted a cross-sectional analysis of a subsample (n = 1,562) of the EPIC-Potsdam study, which involves 27,548 middle-aged men and women. Baseline blood samples have been analyzed for proportions of 32 FAs in erythrocyte membranes (determined by gas chromatography) and biomarker concentrations in plasma. As indicators for DNL, the DNL-index (16:0 / 18:2n-6) and proportions of individual blood FAs in the DNL pathway were used. Plasma parameters associated with liver fat content (fetuin-A, ALT, and GGT) and the algorithm-based fatty liver index (FLI) were used to reflect liver fat accumulation.

**Results:**

The DNL-index tended to be positively associated with the FLI and was positively associated with GGT activity in men (*p* for trend: 0.12 and 0.003). Proportions of 14:0 and 16:0 in erythrocytes were positively associated with fetuin-A, whereas 16:1n-7 were positively associated with the FLI and GGT activity (all *p* for trends in both sexes at least 0.004). Furthermore, the proportion of 16:1n-7 was positively related to fetuin-A in women and ALT activity in men (all *p* for trend at least 0.03). The proportion of 16:1n-9 showed positive associations with the FLI and GGT activity in men and fetuin-A in both sexes, whereas 18:1n-7 was positively associated with GGT activity in men (all *p* for trend at least 0.048).

**Conclusion:**

Findings from this large epidemiological study suggest that liver fat accumulation could link erythrocyte FAs in the DNL pathway to the risk of cardiometabolic diseases.

## Introduction

*De novo* lipogenesis (DNL) is an endogenous pathway in which proteins and carbohydrates are converted to saturated fatty acids (SFA). Fatty acids (FAs) produced by DNL may subsequently be converted to hepatic triglycerides, which accumulate in the liver, when there is an imbalance between DNL and FA uptake, on the one hand, and FA oxidation and very low-density lipoprotein (VLDL) secretion on the other hand [1]. Prospective cohort studies found direct relations between several FA products of DNL in blood and an increased risk of type 2 diabetes [2–9] and cardiovascular disease [10–13]. Liver fat accumulation is considered as a plausible pathway that could link DNL and its FA products to risk of cardiometabolic diseases. Indeed, when estimated using tracer techniques, DNL has been found to be significantly increased in individuals with high liver fat content [14–16]. Previous interventional studies demonstrated that circulating concentrations of specific FAs in the DNL pathway are upregulated in humans by relatively extreme diets [17,18]. Population-based studies consider usual ranges of dietary and lifestyle exposures that influence DNL. Hence, these studies are considered to provide additional important information to intervention studies as they could indicate the relevance under real-world conditions. Data from human population-based studies, which have examined the relations between FA products of DNL and liver fat accumulation, are, however, very limited.

**Fig 1:**
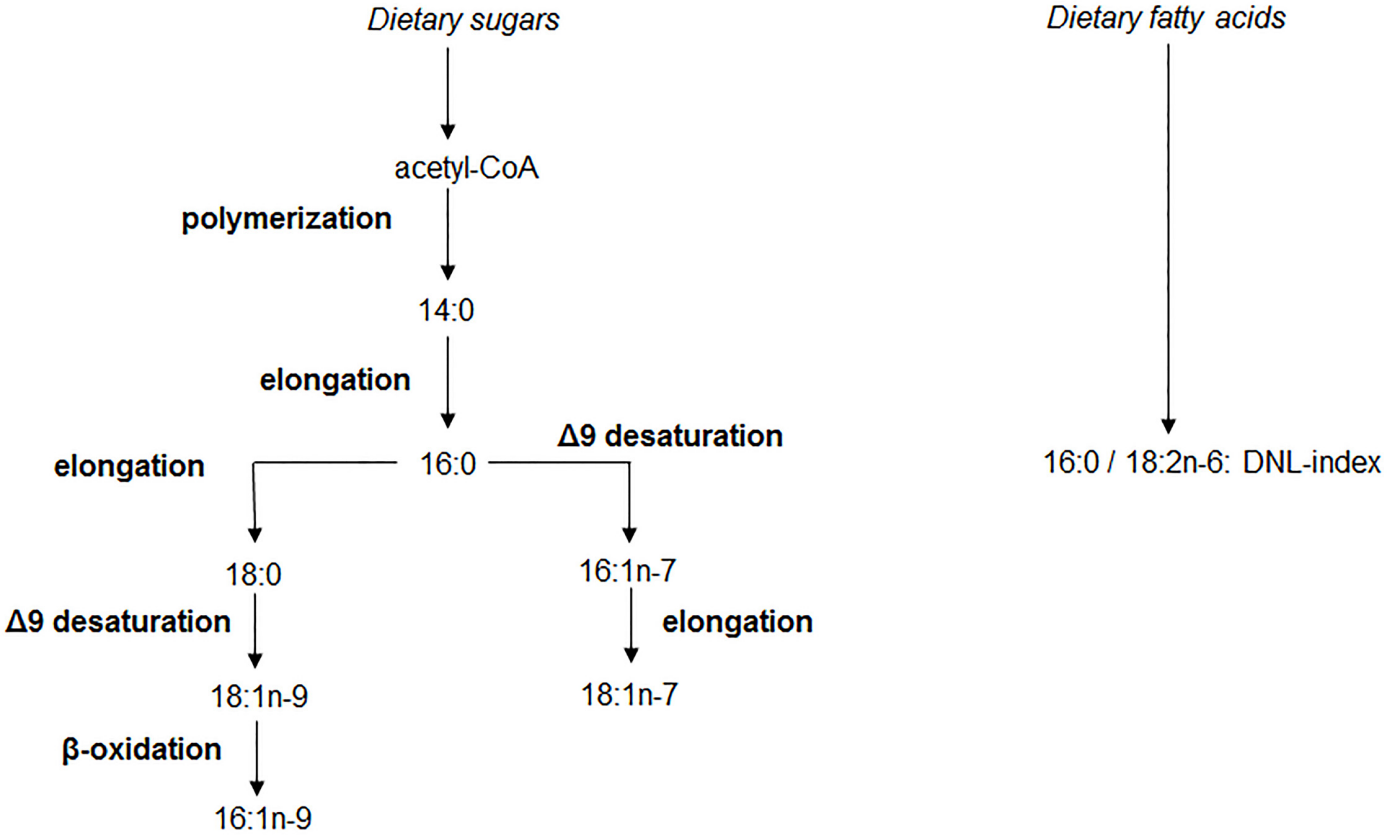
Major products of the DNL pathway. Acetyl-Coenzyme A (acetyl CoA) is polymerized to form FAs. The initial major product of DNL is palmitic acid (16:0) which can be transformed to palmitoleic acid (16:1n-7) by Δ9 desaturation catalyzed by the Stearoyl-Coenzyme A desaturase and subsequently elongated to *cis*-vaccenic acid (18:1n-7) or elongated to stearic acid (18:0) and subsequently desaturated to oleic acid (18:1n-9). Myristic acid (14:0) is another possible minor product of FA synthesis. Experimental studies found that 7-hexadecanoic acid (16:1n-9) could be derived from the β-oxidation of 18:1n-9 [21]. The DNL-index is the ratio of the endogenously produced palmitic acid (16:0), the main product of DNL, and the essential FA linoleic acid (18:2n-6) whose origin is from dietary lipids. The DNL-index has been proposed as a tool to assess FA synthesis in humans [19,20].

DNL is difficult to measure directly, however, its products can be detected in biological samples, such as blood components and adipose tissue [17,19,20]. The main FA products of DNL include palmitic acid (16:0), stearic acid (18:0), palmitoleic acid (16:1n-7), *cis*-vaccenic acid (18:1n-7) and oleic acid (18:1n-9) (**Fig 1**). Furthermore, 7-hexadecanoic acid (16:1n-9) is a FA associated with the DNL pathway, derived from partial β-oxidation of oleic acid [21], and myristic acid (14:0) is a possible minor product of DNL. If substantial DNL occurred, an increase in the proportion of *de novo* synthesized FAs (e.g. palmitate) relative to essential FAs (e.g. linoleate) would be expected. Indeed, intervention studies demonstrated that VLDL-triglycerides [19,20] and erythrocyte membranes [18] were noticeably enriched in palmitate and deficient in linoleate in participants on an isocaloric high-carbohydrate and low-fat diet, which is well known to stimulate DNL, compared to participants on a high-fat, low-carbohydrate [19,20] or a moderate-fat [18] diet. The DNL-index (16:0 / 18:2n-6) has, therefore, been proposed as a tool to assess DNL in humans [19,20].

Liver tissue sampling is not suitable for the measurement of liver fat content in large population-based studies due to invasiveness and related health risks and high costs, whereas imaging techniques are not available in many large cohorts. Population-based studies often use blood parameters that are associated with a metabolically malignant non-alcoholic fatty liver [22] such as the liver enzymes γ-glutamyltransferase (GGT) and alanine transaminase (ALT), or fetuin-A [23], to estimate liver fat content. Fetuin-A has been proposed as one of the most important hepatokines regulating human metabolism [24]. It serves as an adaptor protein for SFAs allowing them to activate the Toll-like receptor 4 which subsequently induces inflammatory signaling [24,25]. Fetuin-A furthermore suppresses the production of the insulin-sensitizing adipokine adiponectin [26], which appears to interact with the transcription factor peroxisome proliferator-activated receptor α leading to increased FA oxidation rate [27] and successively less liver fat accumulation. In addition, algorithm-based indexes such as the “Fatty Liver Index” (FLI), proposed by Bedogni *et al*. [28], have been used to reflect liver fat accumulation. The FLI showed a good accuracy in detecting fatty liver disease [28] and was externally validated [29–35]. In addition, the FLI was shown to predict fatty liver better than liver enzymes [31]. To our knowledge, associations of blood FAs in the DNL pathway with the FLI, as a proxy for liver fat accumulation, and the hepatokine fetuin-A [24], have not been investigated so far. Moreover, studies investigating relations between blood FAs in the DNL pathway [16,36,37] and the DNL-index [16,36,38,39] and liver fat accumulation are scarce. These few studies suggest positive associations [16,36–39].

Therefore, we aimed to study associations of individual erythrocyte FAs in the DNL pathway and the DNL-index with the liver fat blood parameters GGT, ALT and fetuin-A, as well as the FLI, as measures of liver fat accumulation, in a large population-based study.

## Subjects and Methods

### Study population

The European Prospective Investigation into Cancer and Nutrition (EPIC)-Potsdam study is part of the multi-center prospective cohort study EPIC [40]. EPIC-Potsdam includes 16,644 women mainly aged 35 to 64 years and 10,904 men mainly aged 40 to 64 years recruited from the general population of the city of Potsdam, Germany, and surrounding municipalities from 1994 to 1998. Information on education, smoking, and physical activity were assessed at baseline with a self-administered questionnaire and a personal PC-guided interview [40]. The baseline assessment included the collection of blood samples. Anthropometric measurement procedures followed a standardized protocol [40].

For biochemical measurements, a random sample of 2,500 individuals was drawn from all participants of EPIC-Potsdam who provided a blood sample (*n* = 26,444). We excluded individuals with insufficiently filled blood monovettes (*n* = 73), missing or implausible values for erythrocyte FAs (*n* = 651) and for liver and lipid markers (*n* = 136) and participants with intake of lipid-lowering or liver therapeutics or diagnosis of liver cancer (*n* = 78). After exclusion, 1,562 participants were considered for the cross-sectional analysis.

### Ethics Statement

All participants gave their written informed consent and approval was given by the Ethics Committee of the state of Brandenburg, Germany.

### Biochemical Measurements

Thirty milliliters of blood were obtained from each participant during baseline examination, mostly in the non-fasting status. Plasma, serum, red blood cells and buffy coat were stored at -80°C. The erythrocyte membrane FAs were analyzed between February and June 2008. 32 FAs were determined by gas chromatography and expressed as the percentage of total FAs present in the chromatogram [9]. Detailed information with respect to the storage conditions of samples, sample preparation and analytical procedures were described in detail elsewhere [9]. In brief, FA methyl ester (FAME) were separated on a GC-3900 gas chromatograph (Varian Inc, Middelburg, Netherlands) equipped with a 100 m x 0.25mm ID WCOT-fused silica capillary column and flame ionization detector with separation of FAME peaks based on mixed FAME standards (Sigma Aldrich, St Louis, USA). The Galaxie software version 1.9.3.2 (Varian Inc) was used for quantification and identification of peaks. For the erythrocyte membrane FA measurements, intra-assay CVs calculated from a total number of 40 FA measurements in a subset of 20 samples were ≤ 10% for most FAs, with the exception of 16:1n-9 (16.0%).

Plasma levels of triglycerides, GGT, ALT, and fetuin-A were determined with the automatic ADVIA 1650 analyzer (Siemens Medical Solutions, Erlangen, Germany) at the Department of Internal Medicine of the University of Tübingen, Germany, in 2007 [41,42]. All assays were performed according to the manufacturer’s description. Inter-assay CVs for plasma triglycerides were <1.5%, for GGT <2.1%, for ALT <2.5%, and for fetuin-A 5.4% [41]. Plasma concentrations of the biomarkers were multiplied by 1.16 for women and 1.17 for men in order to obtain levels for these citrate plasma samples comparable to levels obtainable from EDTA plasma [43].

### Definition of exposure (DNL)

As mentioned above, FA products of DNL include the FAs 14:0, 16:0, 16:1n-7, 16:1n-9, 18:0, 18:1n-7 and 18:1n-9 (**Fig 1**). Prior studies have shown that the proportion of 18:0 in erythrocytes was not increased after a low-fat, high-carbohydrate diet [18] which is well known to stimulate the DNL. Furthermore, endogenously synthesized 18:1n-9 may be diluted due to the high intake of 18:1n-9 in the diet (median (IQR) of dietary 18:1n-9 intake in the EPIC-Potsdam subcohort was 29.3 (28.3–30.5)% of total fat intake [9]). Therefore, the proportion of 18:1n-9 in erythrocytes was not considered in this analysis. To focus on FAs that more strongly reflect activated DNL, the proportions of 14:0, 16:0, 16:1n-7, 16:1n-9, and 18:1n-7 in erythrocyte membranes, that increased in response to a low-fat diet [18], were chosen as exposure of the seven potential FAs. The DNL-index (16:0 / 18:2n-6) was also considered as exposure.

### Statistical analysis

We performed multivariable linear regression analysis to investigate the relations of erythrocyte FAs to fetuin-A, ALT, GGT concentrations and the FLI according to Bedogni *et al*. [28] which was calculated based on this formula:

$$FLI\;=\;\frac{e^{\left(0.953\;*\;ln\left(TG\right)\;+\;0.139\;*\;BMI\;+\;0.718\;*\;ln\left(GGT\right)\;+\;0.053\;*\;WC\;-\;15.745\right)}}{1+e^{\left(0.953\;*\;ln\left(TG\right)\;+\;0.139\;*\;BMI\;+\;0.718\;*\;ln\left(GGT\right)\;+\;0.053\;*\;WC\;-\;15.745\right)}}*100$$

(GGT in U/L, triglycerides (TG) in mg/dL, waist circumference (WC) in cm and body mass index (BMI) in kg/m^2^)

Categorical variables were entered as binary indicator variables into the models. We tested for interaction with sex by evaluating statistical significance of cross-product terms of the respective FAs / FA-ratio (as a quantitative variable) and sex included in the multivariable-adjusted models. We detected significant interaction terms with sex for a number of associations (the DNL-index with GGT, 14:0 with ALT and GGT, 16:1n-7 with ALT, GGT and the FLI, and 18:1n-7 with GGT). Therefore, analyses were performed stratified by sex.

We used the log_e_ transformation of ALT, GGT and the FLI to normalize the right skewed distribution. We modeled the individual FA proportions and the FA-ratio as tertiles to account for nonlinear relations with the outcomes. We estimated geometric means and 95% confidence intervals (CI) in case of ALT, GGT and the FLI and arithmetric means and 95% CI in case of fetuin-A by FA tertiles and tested for statistical significance of linear trends across tertiles by modeling the median value of the FA within each tertile as a quantitative variable. We calculated a multivariable-adjusted model adjusted for smoking status (never, past, current <20 units/day, current ≥20 units/day), education status (in or no training, vocational training, technical school, technical college or university degree), alcohol intake (0, >0–6; >6–12; >12–24; >24–60; >60–96; >96 g/d), leisure time sports activity (no sports, ≤4 h/week, >4 h/week), biking (no biking, <2.5 h/week, 2.5–4.9 h/week, ≥5 h/week), hormone use in women (none, oral contraceptive, hormone replacement therapy [HRT]), percentage of energy intake from the sum of mono- and disaccharides, percentage of energy intake from polysaccharides, percentage of energy intake from fat, BMI and waist circumference. The presence of colinearity among independent variables was tested measuring the variance inflation factor in linear regression analysis.

In addition, we performed sensitivity analyses to assess the robustness of our results: Because the fasting status may influence triglyceride concentration, which is a component of the FLI, we examined only fasted participants (*n* = 215) in a first sensitivity analysis. Fasting was defined as time of the last meal or drink >8 h. Because we assumed that the diagnosis of a chronic disease may have changed the participants eating behavior, we further excluded participants with a history of cancer, diabetes or cardiovascular diseases in a second sensitivity analysis, leaving 1,391 participants, 514 men and 877 women, for analyses. Excessive alcohol consumption is known to lead to alcoholic fatty liver disease. The focus of this analysis was however on non-alcoholic fatty liver accumulation. Therefore, in a third sensitivity analysis, we excluded men with a consumption of >30 g alcohol per day and women with a consumption of >20 g alcohol per day [44], leaving 1,309 participants for analysis (426 men, 883 women). We also investigated the impact of high lifetime alcohol consumption on the results by excluding participants who were former heavy drinkers or consumed alcohol occasionally heavy or always heavy during their lifetime [45] in a fourth sensitivity analysis, leaving 1,383 participants for analysis (463 men, 920 women). Additionally, we performed a fifth sensitivity analysis with exclusion of participants with HbA1c values ≥5.7% to investigate the possibility of reverse causation due to a pre-diabetic state (785 women, 430 men remained).

The type I error for each FA model was set to 5 percent. All statistical tests were two tailed and 95% CI were estimated. A *p* for trend value of 0.00833 and below would satisfy a correction for multiple testing according to Bonferroni (0.05 / 6 comparisons), if one would consider our analysis as completely exploratory. We performed the statistical analysis with the SAS software, release 9.4 (SAS Institute Inc., Cary, NC, USA).

## Results

The characteristics of the study population by tertiles of the DNL-index and proportions of palmitic acid and palmitoleic acid in erythrocyte membranes, stratified by sex, are shown in **Tables 1** and **2**. Participants with a high proportion of palmitoleic acid and DNL-index were more obese, had a higher waist circumference and a lower energy intake from fat compared to others. Participants with high proportions of palmitic acid and palmitoleic acid and a high DNL-index also consumed more alcohol compared to other participants.

**Table 1:** Characteristics by tertiles of the DNL-index (16:0 / 18:2n-6), palmitic acid (16:0) and palmitoleic acid (16:1n-7) in men, EPIC-Potsdam study (n = 573) ^a^.

	DNL-index			Palmitic acid			Palmitoleic acid		
	Tertile 1	Tertile 3	*p* value	Tertile 1	Tertile 3	*p* value	Tertile 1	Tertile 3	*p* value
Age (y)	50.8 (15.9)	51.9 (15.4)	0.27	50.7 (14.6)	52.7 (15.1)	0.69	51.4 (15.2)	53.0 (14.5)	0.30
BMI (kg/m^2^)	25.6 (4.33)	27.3 (5.22)	0.0001	26.1 (4.28)	26.0 (4.53)	0.83	25.7 (3.79)	27.5 (5.17)	<0.0001
Waist circumference (cm)	90.3 (11.0)	94.5 (13.0)	<0.0001	92.5 (13.0)	92.0 (13.0)	0.75	90.0 (10.5)	96.0 (14.5)	<0.0001
Leisure time sports activity (h/week)	0.00 (1.00)	0.00 (2.00)	0.24	0.00 (1.50)	0.00 (1.50)	0.62	0.00 (2.00)	0.00 (1.50)	0.07
Biking (h/week)	1.00 (2.50)	0.50 (2.00)	0.28	0.50 (2.50)	1.00 (2.50)	0.30	1.00 (2.50)	0.50 (2.00)	0.11
Smoking			0.21			0.05			0.54
Never smoker (%)	33.0	24.6		34.6	25.1		29.3	26.2	
Education			0.78			0.24			0.40
Technical college, university (%)	49.7	48.7		48.2	51.3		55.5	48.2	
Alcohol intake (g/day)	12.7 (19.1)	23.3 (32.4)	<0.0001	14.8 (18.8)	20.6 (30.0)	0.0003	11.9 (17.9)	25.2 (32.3)	<0.0001
Energy intake from mono- and disaccharides (%)	19.1 (7.37)	16.7 (7.85)	0.0007	18.2 (7.91)	18.1 (7.29)	0.27	18.1 (8.25)	16.8 (8.36)	0.02
Energy intake from polysaccharides (%)	24.5 (6.17)	22.1 (6.91)	<0.0001	24.4 (5.95)	22.8 (6.04)	0.007	24.3 (6.14)	21.9 (6.44)	<0.0001
Energy intake from fat (%)	44.8 (8.58)	43.7 (8.61)	0.045	44.8 (8.98)	44.0 (8.46)	0.33	45.1 (8.65)	43.3 (10.3)	0.013

^a^ For continuous variables, values are medians (IQR); for categorical variables, values are percentages (all such values). *P* value reflects whether the values of the variables significantly differ between extreme erythrocyte FA/FA-ratio tertiles (Wilcoxon’s test for continuous variables and χ2 test for categorical variables).

**Table 2:** Characteristics by tertiles of the DNL-index (16:0 / 18:2n-6), palmitic acid (16:0) and palmitoleic acid (16:1n-7) in women, EPIC-Potsdam study (n = 989) ^a^.

	DNL-index			Palmitic acid			Palmitoleic acid		
	Tertile 1	Tertile 3	*p* value	Tertile 1	Tertile 3	*p* value	Tertile 1	Tertile 3	*p* value
Age (y)	45.2 (16.3)	49.0 (17.2)	0.002	49.6 (15.6)	45.8 (16.8)	0.001	45.6 (15.2)	49.3 (16.1)	0.0001
BMI (kg/m^2^)	24.2 (5.84)	25.9 (5.30)	0.0001	24.8 (5.40)	24.3 (5.39)	0.23	24.1 (4.70)	25.8 (6.27)	<0.0001
Waist circumference (cm)	77.0 (15.0)	80.3 (15.5)	0.0002	79.0 (15.5)	76.3 (14.0)	0.14	76.0 (13.0)	81.0 (17.5)	<0.0001
Leisure time sports activity (h/week)	0.00 (1.50)	0.00 (2.00)	0.47	0.00 (1.00)	0.00 (2.00)	0.0006	0.00 (2.00)	0.00 (1.00)	0.07
Biking (h/week)	1.00 (3.00)	1.00 (2.50)	0.12	0.50 (2.50)	1.00 (3.00)	0.012	0.50 (3.00)	0.50 (2.00)	0.10
Smoking			0.50			0.01			0.03
Never smoker (%)	60.2	56.1		63.5	53.0		62.9	52.4	
Education			0.26			0.42			0.63
Technical college, university (%)	30.7	33.6		28.9	32.1		31.3	31.2	
Oral contraceptive use (%)	14.3	17.9	0.21	8.21	27.0	<0.0001	11.6	21.8	0.0004
Hormone replacement therapy (%)	21.6	23.9	0.47	26.1	22.7	0.31	23.7	22.4	0.70
Alcohol intake (g/day)	3.74 (6.87)	6.23 (12.4)	<0.0001	4.36 (8.44)	5.80 (9.14)	0.003	4.59 (7.49)	5.66 (11.1)	0.004
Energy intake from mono- and disaccharides (%)	22.6 (8.60)	23.1 (9.47)	0.82	22.8 (8.55)	22.6 (8.24)	0.68	22.5 (8.58)	23.3 (8.74)	0.06
Energy intake from polysaccharides (%)	23.4 (6.48)	21.6 (7.19)	<0.0001	22.7 (6.18)	23.0 (7.06)	0.91	23.0 (6.20)	22.7 (7.09)	0.05
Energy intake from fat (%)	37.2 (5.78)	36.2 (6.63)	0.014	37.1 (6.27)	36.4 (5.77)	0.02	37.2 (5.69)	36.0 (6.43)	<0.0001

^a^ For continuous variables, values are medians (IQR); for categorical variables, values are percentages (all such values). *P* value reflects whether the values of the variables significantly differ between extreme erythrocyte FA/FA-ratio tertiles (Wilcoxon’s test for continuous variables and χ2 test for categorical variables).

### Associations between DNL and liver markers and the FLI

**Table 3** shows multivariable-adjusted means and 95% CIs of the FLI, GGT, ALT and fetuin-A according to tertiles of erythrocyte FAs and FA-ratio, stratified by sex. The DNL-index (16:0 / 18:2n-6) tended to be positively associated with the liver enzymes and the FLI in men and fetuin-A concentration in both sexes, reaching significance only with GGT activity in men. Regarding SFAs, the proportion of 14:0 was positively associated with the FLI in men and fetuin-A concentrations in both sexes, and the proportion of 16:0 showed positive associations with fetuin-A, whereas no clear associations were detected with other liver markers. Positive associations were further seen between the MUFAs 16:1n-9 (in men only) and 16:1n-7 and the FLI as well as GGT activity. A positive trend was also detected between 16:1n-7 (in women only) and 16:1n-9 and fetuin-A, and between the proportion of 16:1n-7 in erythrocytes and ALT activity in men. The proportion of 18:1n-7 in erythrocytes was positively associated with the FLI and GGT activity in men, reaching significance only with GGT activity, but showed no associations in women.

**Table 3:** Adjusted geometric means of the fatty liver index (FLI), plasma GGT and ALT and adjusted arithmetic means (95% CI) of plasma fetuin-A by tertiles of erythrocyte FA proportions for men (n = 573) and women (n = 989), EPIC-Potsdam study ^a^.

	Men				Women			
	Tertile of fatty acid			*p* for trend	Tertile of fatty acid			*p* for trend
	1	2	3		1	2	3	
16:0 / 18:2n-6 (DNL-index)								
FLI [Score points]	37.9 (35.2–40.8)	39.7 (37.0–42.7)	41.3 (38.4–44.4)	0.12	12.0 (11.2–12.9)	12.6 (11.8–13.5)	12.5 (11.7–13.4)	0.43
GGT [μkat/l]	0.43 (0.39–0.48)	0.49 (0.44–0.55)	0.55 (0.49–0.61)	0.003	0.23 (0.21–0.25)	0.24 (0.22–0.26)	0.24 (0.22–0.26)	0.66
ALT [μkat/l]	0.44 (0.41–0.47)	0.47 (0.44–0.50)	0.49 (0.45–0.52)	0.06	0.29 (0.27–0.30)	0.29 (0.28–0.30)	0.29 (0.28–0.30)	0.99
fetuin-A [μg/ml]	253 (245–261)	260 (252–268)	262 (254–270)	0.15	264 (257–271)	261 (254–268)	273 (266–280)	0.06
14:0								
FLI [Score points]	38.1 (35.4–40.9)	37.0 (34.5–39.7)	44.2 (41.1–47.4)	0.003	12.1 (11.3–12.9)	12.2 (11.4–13.0)	13.0 (12.1–13.9)	0.14
GGT [μkat/l]	0.48 (0.43–0.53)	0.45 (0.41–0.50)	0.54 (0.48–0.60)	0.12	0.24 (0.22–0.26)	0.23 (0.21–0.25)	0.24 (0.23–0.26)	0.71
ALT [μkat/l]	0.45 (0.42–0.48)	0.46 (0.43–0.49)	0.48 (0.45–0.52)	0.17	0.29 (0.28–0.31)	0.29 (0.27–0.30)	0.28 (0.27–0.30)	0.22
fetuin-A [μg/ml]	253 (245–261)	254 (246–262)	268 (260–275)	0.009	256 (249–262)	264 (258–271)	278 (271–284)	<0.0001
16:0								
FLI [Score points]	38.7 (36.0–41.6)	40.5 (37.7–43.5)	39.8 (37.0–42.7)	0.60	11.9 (11.2–12.8)	12.7 (11.9–13.6)	12.6 (11.8–13.5)	0.30
GGT [μkat/l]	0.48 (0.43–0.53)	0.49 (0.44–0.54)	0.50 (0.45–0.56)	0.54	0.23 (0.21–0.25)	0.24 (0.22–0.26)	0.24 (0.22–0.26)	0.47
ALT [μkat/l]	0.46 (0.43–0.49)	0.47 (0.44–0.51)	0.47 (0.44–0.50)	0.65	0.30 (0.28–0.31)	0.29 (0.28–0.30)	0.28 (0.27–0.29)	0.07
fetuin-A [μg/ml]	250 (243–258)	255 (247–262)	270 (262–278)	0.0006	257 (250–264)	261 (254–267)	280 (274–287)	<0.0001
16:1n-7								
FLI [Score points]	35.6 (33.1–38.2)	37.7 (35.2–40.5)	46.4 (43.1–49.9)	<0.0001	11.0 (10.3–11.8)	12.1 (11.3–12.9)	14.3 (13.3–15.3)	<0.0001
GGT [μkat/l]	0.45 (0.41–0.50)	0.41 (0.37–0.45)	0.63 (0.57–0.70)	<0.0001	0.22 (0.20–0.24)	0.23 (0.22–0.25)	0.26 (0.24–0.28)	0.004
ALT [μkat/l]	0.45 (0.42–0.48)	0.43 (0.41–0.46)	0.52 (0.48–0.55)	0.004	0.29 (0.27–0.3)	0.28 (0.27–0.29)	0.30 (0.28–0.31)	0.25
fetuin-A [μg/ml]	261 (253–269)	258 (251–266)	255 (247–264)	0.36	260 (254–267)	266 (259–273)	271 (265–278)	0.03
16:1n-9								
FLI [Score points]	36.6 (34.0–39.3)	39.7 (37.0–42.7)	42.8 (39.9–46.0)	0.003	12.3 (11.5–13.2)	12.4 (11.6–13.2)	12.5 (11.7–13.4)	0.70
GGT [μkat/l]	0.45 (0.40–0.50)	0.49 (0.44–0.54)	0.53 (0.48–0.59)	0.02	0.23 (0.21–0.25)	0.24 (0.22–0.26)	0.24 (0.22–0.26)	0.55
ALT [μkat/l]	0.45 (0.42–0.48)	0.47 (0.44–0.50)	0.48 (0.45–0.51)	0.25	0.29 (0.28–0.31)	0.29 (0.28–0.31)	0.28 (0.27–0.29)	0.06
fetuin-A [μg/ml]	249 (241–257)	264 (256–271)	262 (254–270)	0.048	256 (249–262)	271 (265–278)	271 (264–277)	0.007
18:1n-7								
FLI [Score points]	37.3 (34.7–40.1)	40.8 (38.0–43.9)	40.8 (38.0–43.9)	0.09	12.4 (11.5–13.2)	12.3 (11.5–13.1)	12.6 (11.7–13.5)	0.74
GGT [μkat/l]	0.45 (0.40–0.50)	0.47 (0.43–0.53)	0.55 (0.49–0.61)	0.008	0.23 (0.21–0.25)	0.23 (0.21–0.25)	0.25 (0.23–0.27)	0.16
ALT [μkat/l]	0.46 (0.43–0.49)	0.46 (0.43–0.49)	0.48 (0.45–0.51)	0.48	0.29 (0.28–0.30)	0.28 (0.27–0.29)	0.30 (0.28–0.31)	0.47
fetuin-A [μg/ml]	258 (250–266)	254 (247–262)	263 (255–270)	0.41	264 (257–270)	264 (258–271)	270 (263–277)	0.21

^a^ In a multivariable linear regression analysis, we modeled the individual FA proportions as tertiles. The model was adjusted for age at recruitment, smoking status (never, past, current smoker <20 units/days, current smoker ≥20 units/days), alcohol intake (0, >0–6; >6–12; >12–24; >24–60; >60–96; >96 g/d), leisure time sports activity (no sports, ≤4 h/week, >4 h/week), biking (no biking, <2.5 h/week, 2.5–4.9 h/week, ≥5 h/week), hormone use in women (none, oral contraceptive, hormone replacement therapy [HRT]), education status (in or no training, vocational training, technical school, technical college or university degree), energy intake from the sum of mono- and disaccharides (%), energy intake from polysaccharides (%), energy intake from fat (%), BMI (kg/m^2^) and waist circumference (cm). We estimated geometric means and 95% confidence intervals (CI) in case of GGT, ALT and the FLI and arithmetic means and 95% CI in case of fetuin-A by FA tertiles and tested for statistical significance of linear trends across FA tertiles by modeling the median value of the FA within each tertile as a quantitative variable. *P* for trend value reflects whether the biomarker significantly increases or decreases across the FA tertiles.

These following associations satisfy *p* for trend values according to correction for multiple testing if one considered our analysis as exploratory: the DNL-index with GGT in men only, 14:0 with the FLI in men and fetuin-A in women, 16:0 with fetuin-A in both sexes, 16:1n-7 with ALT in men, and with the FLI and GGT in both sexes, 16:1n-9 with the FLI in men and fetuin-A in women, and 18:1n-7 with GGT in men. We considered these associations as our main findings.

### Sensitivity analyses

In general, after restricting analyses to fasted participants (**S1 Table**), associations were qualitatively comparable, particularly in women, however, only few reached statistical significance due to the loss of power. The exclusion of prevalent cases of diabetes, cardiovascular disease and cancer (**S2 Table**), participants with exceeding alcohol consumption at baseline (**S3 Table**) or exceeding lifetime alcohol consumption (**S4 Table**) or participants with HbA1c values ≥5.7% (**S5 Table**) had no major influence on the results.

## Discussion

In this cross-sectional study of middle-aged men and women, proportions of FAs in erythrocyte membranes in the DNL pathway and the DNL-index, reflecting the dilution of essential FAs by those synthesized *de novo*, were positively associated with proxies of liver fat accumulation. Most notably, the DNL-index was positively related to GGT activity in men and proportions of 14:0 and 16:0 in erythrocytes were positively associated with fetuin-A concentrations, whereas the proportion of 16:1n-7 showed positive associations with GGT activity and the algorithm-derived FLI, the proportion of 16:1n-9 was directly associated with fetuin-A in both sexes and with GGT activity and the FLI in men, and the proportion of 18:1n-7 in erythrocytes was positively related to GGT activity in men.

Our observation that erythrocyte FAs in the DNL pathway and the DNL-index are linked to proxies of liver fat accumulation is in line with previous studies. In a case-control study on histologically determined nonalcoholic fatty liver among severely obese persons, the DNL-index, estimated from FA-ratios derived from hepatic tissue, was positively correlated to liver fat content [38]. A cross-sectional study in Swedish elderly men suggested positive associations between the DNL-index and the proportion of 16:1n-7 in serum cholesterol esters and ALT activity [36]. Furthermore, a cross-sectional analysis in middle-aged and older Chinese found that proportions of 14:0, 16:1n-7, and 16:1n-9 in erythrocytes were significantly positively correlated with ALT and GGT activity, however 16:0 and 18:1n-7 showed no significant correlations after multivariable-adjustment [37]. The heterogeneity of the above-mentioned studies and our study e.g. with respect to study designs, study populations, genetic background, lifestyle and eating habits, adjustment set and fat compartments complicates comparison of the results. Regarding different adjustment sets across studies, crude correlation coefficients were calculated in the case-control study [38], whereas the models in the cross-sectional study in Swedish elderly men were adjusted for alcohol intake, physical activity, abdominal obesity and insulin sensitivity [36], and the models in the cross-sectional study in middle-aged and older Chinese were adjusted for age, sex, region and residence [37]. Results from our study suggest that the observed associations are also largely independent of other nutritional and lifestyle factors. With respect to the use of different fat compartments across studies, other studies used the DNL-index and FAs in the DNL pathway based on used serum cholesterol esters [36], unfractionated lipid fraction [38] or, in accordance with our study, phospholipids from erythrocyte membranes [37]. Whereas the use of unfractionated lipids may be difficult to interpret, results from randomized controlled trials indicate a certain transferability of results of DNL-indexes as well as FAs in the DNL pathway derived from different lipid fractions. It was demonstrated that the proportions of 14:0, 16:0, 16:1n-7, 16:1n-9, and 18:1n-7 were significantly increased, whereas the proportion of 18:2n-6 was significantly decreased in plasma cholesterol esters and erythrocyte membranes in participants on a low-fat, high-carbohydrate diet compared to individuals on a moderate-fat diet [18]. In both lipid fractions, particularly the proportions of 16:1n-7 and 16:1n-9 increased in participants on a low-fat, high-carbohydrate diet (change compared to baseline: erythrocyte membranes 32.2 and 26.8%, cholesterol esters 34.4 and 33.2%, respectively), whereas the proportions of 14:0, 16:0, and 18:1n-7 increased to a lower extent (change compared to baseline: erythrocyte membranes 2.7–8.7%, cholesterol esters 11.4–20.8%) [18]. The increase in 16:0 and the depletion of 18:2n-6 was also shown in VLDL-triglycerides in participants on a low-fat compared to participants on a high-fat diet [19,20]. These results could also be interpreted as an indirect measure of a good validity of the DNL-index and FAs in the DNL pathway derived from these different lipid fractions. In line with these findings, the hepatic mRNA expression of lipogenic genes (such as Acetyl-Coenzyme A Carboxylase α) was positively correlated with the DNL-index derived from VLDL-triglycerides (correlation coefficient: 0.62, *p* = 0.01) [46]. Applying this index from VLDL-triglycerides in an interventional study involving healthy subjects, the DNL-index showed a close positive correlation with liver fat content measured by localized ^1^H- magnetic resonance spectroscopy, as well as with changes, thereof, in response to a dietary sugar supplementation [39]. Consistent with findings from observational studies, when estimated using tracer methods, FAs in the DNL pathway were significantly increased in individuals with high liver fat compared to individuals with low liver fat [15,16]. Our findings indicate that erythrocyte FAs in the DNL pathway and the DNL-index are linked to proxies of liver fat accumulation.

Erythrocyte FAs reflecting higher DNL might indicate both, influences of metabolism and dietary intake of FAs. In this regard, a previous analysis in EPIC-Potsdam found that proportions of 14:0, 16:0, 18:0 and 16:1n-7 in erythrocytes correlated only weakly with their estimated dietary intake (no information available for 16:1n-9 and 18:1n-7) [9], possibly due to the fact that dietary SFAs may be variably oxidized after ingestion for energy use [47]. Blood concentrations of FAs in the DNL pathway in our study may therefore rather reflect endogenous synthesis. Intervention studies demonstrated that DNL is stimulated by increased intake of dietary carbohydrates, decreased intake of dietary fat [17,18,20] and increased alcohol consumption [48]. In line with these facts, participants of our study with high DNL markers were more likely to have a higher alcohol intake and a lower energy intake from fat compared to others, whereas energy intake from carbohydrate fractions was not increased as expected from findings from intervention studies. For interpretation of these results it should be taken into account that generally a high carbohydrate intake was used to induce DNL in intervention studies (e.g. 75% energy intake from carbohydrates [20]), which was not reached in our study population (mean energy intake from absorbable carbohydrates: 41%). By adjusting for potential confounding dietary factors, we confirmed the independence of our observed associations.

To our knowledge, our study is the first to investigate associations of blood FAs in the DNL pathway and the DNL-index with the algorithm-based FLI. Whereas intervention studies recommend the DNL-index (16:0 / 18:2n-6) as a tool to assess DNL in humans [19,20], the FLI was shown to predict fatty liver better than liver enzymes [31]. Our analysis therefore provides additional information compared to other studies that solely investigated individual FAs in the DNL pathway or liver markers. A limitation of our study lies in the fact that we used liver markers and the FLI as proxies for liver fat and did not measure liver fat directly via histology or imaging techniques. Large-scaled studies with more direct measures of liver fat and DNL are warranted for confirmation of our results. However, serum fetuin-A concentrations, ALT and GGT activity have been associated with liver fat accumulation, even independently of BMI [49–51]. The FLI showed a good predictive accuracy of fatty liver disease (AUC ROC 0.85 (0.81–0.88)) [28] and was externally validated [29–35]. However, the FLI was created for the purpose to determine occurrence of fatty liver disease and its suitability for estimating percentage of liver fat is unclear. To minimize confounding, we adjusted models including the FLI for BMI and waist circumference. This might have led to an underestimation of the associations, because BMI and waist circumference are components of the FLI. It is a limitation of our study that many of our participants had not fasted before blood drawl. As a component of the FLI score, plasma triglyceride levels are known to depend on food intake. Still, on the population level, triglyceride levels increased only modestly in response to normal food intake [52]. Because the fasting status does not affect the erythrocyte FA profile, at the most, an attenuation of the effect estimates in models including the FLI would be expected. In our sensitivity analysis restricted to fasted participants, associations regarding the FLI were generally qualitatively comparable, though only few reached statistical significance due to the loss of power. As a result of the cross-sectional design, it cannot be determined, whether the erythrocyte FAs and the FA-ratio were the cause or the result of a change in liver markers or FLI (reverse causation); therefore, our analysis should rather be considered as hypothesis-generating. Still, our sensitivity analysis in which we excluded participants with HbA1c values ≥5.7% led to comparable results. We measured DNL indirectly as proportions of erythrocyte FAs reflecting higher DNL and did not measure DNL directly, e.g. using tracer studies. However, the direct measurement of DNL by tracer studies would not be feasible in a cohort as large as ours. Given that the proportions of 14:0, 16:0, 16:1n-7, 16:1n-9, and 18:1n-7 in erythrocyte membranes increased in response to a low-fat diet [18], that is well known to stimulate DNL, the erythrocyte fraction should however reflect stimulated DNL. Erythrocyte FAs were expressed as percentages of total FAs. Consequently, it is difficult to interpret results for individual FAs independent of the other FAs. Proportions of FAs however tend to be more meaningful in terms of interpretation of metabolic relationships than do absolute measurements [53]. Absolute amounts of FAs correlate with the total lipoprotein content, which is related to liver fat accumulation. To avoid these confounding problems, we decided to use the relative proportions of FAs in our study. Random measurement error in erythrocyte FAs and liver markers might have led to an attenuation of the effect estimates.

In conclusion, we observed direct associations of FAs in the DNL pathway and the DNL-index with liver markers and FLI score points. Our findings in this population-based study support the hypothesis that FA products of DNL may be linked to risk of cardiometabolic diseases by modulation of liver fat accumulation.

## Supporting Information

S1 Table Adjusted geometric means of the fatty liver index (FLI), plasma GGT and ALT and adjusted arithmetic means (95% CI) of plasma fetuin-A by tertiles of erythrocyte FA proportions for fasted men (n = 85) and women (n = 130), EPIC-Potsdam study. (DOCX)

S2 Table Adjusted geometric means of the fatty liver index (FLI), plasma GGT and ALT and adjusted arithmetic means (95% CI) of plasma fetuin-A by tertiles of erythrocyte FA proportions, EPIC-Potsdam study. Participants with a history of cancer, diabetes or cardiovascular diseases were excluded leaving 514 men and 877 women for this analysis.
(DOCX)

S3 Table Adjusted geometric means of the fatty liver index (FLI), plasma GGT and ALT and adjusted arithmetic means (95% CI) of plasma fetuin-A by tertiles of erythrocyte FA proportions, EPIC-Potsdam study. Men with a consumption of >30 g alcohol per day and women with a consumption of >20 g alcohol per day were excluded leaving 426 men and 883 women for this analysis.
(DOCX)

S4 Table Adjusted geometric means of the fatty liver index (FLI), plasma GGT and ALT and adjusted arithmetic means (95% CI) of plasma fetuin-A by tertiles of erythrocyte FA proportions, EPIC-Potsdam study. Participants who were former heavy drinkers or consumed alcohol occasionally heavy or always heavy during their lifetime were excluded leaving 463 men and 920 women for this analysis.
(DOCX)

S5 Table Adjusted geometric means of the fatty liver index (FLI), plasma GGT and ALT and adjusted arithmetic means (95% CI) of plasma fetuin-A by tertiles of erythrocyte FA proportions, EPIC-Potsdam study. Participants with HbA1c values ≥5.7% were excluded leaving 430 men and 785 women for this analysis. (DOCX)
