# Supplementary material for: Associations of Erythrocyte Fatty Acids in the De Novo Lipogenesis Pathway with Proxies of Liver Fat Accumulation in the EPIC-Potsdam Study
Source: PLoS One. 2015 May 18;10(5):e0127368. doi: 10.1371/journal.pone.0127368 (PMC4435749; doi:10.1371/journal.pone.0127368)
Supplement: S1 Table — (DOCX) [file pone.0127368.s001.docx]

Table S1. Adjusted geometric means of the fatty liver index (FLI), plasma GGT and ALT and adjusted arithmetic means (95% CI) of plasma fetuin-A by tertiles of erythrocyte FA proportions for fasted men (n=85) and women (n=130), EPIC-Potsdam study ^a^.

|  | | Men | | | | Women | | | |
| --- | --- | --- | --- | --- | --- | --- | --- | --- | --- |
|  | | Tertile of fatty acid | | | *p* for trend | Tertile of fatty acid | | | *p* for  trend |
|  | | 1 | 2 | 3 |  | 1 | 2 | 3 |  |
| 16:0 / 18:2n-6 (DNL-index) | | |  |  |  |  |  |  |  |
| FLI [Score points] | 33.8 (27.5-41.5) | | 47.9 (40.2-57.2) | 41.5 (33.4-51.6) | 0.22 | 11.4 (9.31-14.0) | 14.9 (12.1-18.2) | 13.1 (10.7-16.1) | 0.45 |
| GGT [μkat/l] | 0.42 (0.30-0.58) | | 0.76 (0.57-1.00) | 0.64 (0.45-0.90) | 0.12 | 0.19 (0.15-0.24) | 0.27 (0.21-0.34) | 0.28 (0.22-0.35) | 0.05 |
| ALT [μkat/l] | 0.47 (0.39-0.56) | | 0.48 (0.41-0.56) | 0.47 (0.39-0.57) | 0.99 | 0.28 (0.24-0.32) | 0.30 (0.26-0.34) | 0.34 (0.30-0.39) | 0.04 |
| fetuin-A [μg/ml] | 266 (242-290) | | 267 (247-288) | 257 (232-283) | 0.67 | 270 (250-290) | 270 (250-289) | 285 (265-305) | 0.29 |
|  |  | |  |  |  |  |  |  |  |
| 14:0 |  | |  |  |  |  |  |  |  |
| FLI [Score points] | 40.5 (33.6-48.9) | | 39.0 (32.5-46.9) | 42.8 (35.5-51.5) | 0.66 | 12.0 (9.94-14.5) | 15.2 (12.6-18.3) | 12.2 (10.1-14.8) | 0.98 |
| GGT [μkat/l] | 0.56 (0.42-0.76) | | 0.54 (0.41-0.72) | 0.68 (0.50-0.90) | 0.37 | 0.21 (0.17-0.26) | 0.30 (0.24-0.37) | 0.23 (0.18-0.28) | 0.75 |
| ALT [μkat/l] | 0.48 (0.41-0.56) | | 0.46 (0.40-0.54) | 0.48 (0.41-0.56) | 0.91 | 0.31 (0.27-0.35) | 0.28 (0.25-0.32) | 0.33 (0.29-0.37) | 0.43 |
| fetuin-A [μg/ml] | 265 (244-287) | | 265 (244-285) | 260 (240-281) | 0.74 | 265 (246-283) | 276 (257-294) | 283 (264-302) | 0.18 |
|  |  | |  |  |  |  |  |  |  |
| 16:0 |  | |  |  |  |  |  |  |  |
| FLI [Score points] | 44.7 (37.0-54.0) | | 42.1 (35.2-50.3) | 35.9 (29.8-43.3) | 0.12 | 13.3 (10.9-16.3) | 12.3 (10.1-15.0) | 13.6 (11.2-16.6) | 0.81 |
| GGT [μkat/l] | 0.58 (0.43-0.79) | | 0.57 (0.43-0.76) | 0.62 (0.46-0.83) | 0.78 | 0.23 (0.18-0.29) | 0.23 (0.18-0.29) | 0.26 (0.21-0.33) | 0.42 |
| ALT [μkat/l] | 0.52 (0.44-0.61) | | 0.46(0.39-0.53) | 0.45 (0.38-0.53) | 0.25 | 0.30 (0.27-0.34) | 0.30 (0.26-0.34) | 0.32 (0.28-0.36) | 0.59 |
| fetuin-A [μg/ml] | 270 (249-292) | | 265 (244-285) | 256 (235-277) | 0.37 | 269 (249-288) | 274 (255-294) | 281 (261-300) | 0.40 |
|  |  | |  |  |  |  |  |  |  |
| 16:1n-7 |  | |  |  |  |  |  |  |  |
| FLI [Score points] | 42.5 (35.0-51.6) | | 34.3 (28.7-41.1) | 46.6 (38.0-57.2) | 0.42 | 13.0 (10.8-15.8) | 11.7 (9.68-14.2) | 14.6 (12.0-17.9) | 0.40 |
| GGT [μkat/l] | 0.55 (0.41-0.74) | | 0.43 (0.33-0.57) | 0.86 (0.63-1.18) | 0.04 | 0.24 (0.19-0.30) | 0.21 (0.17-0.26) | 0.28 (0.22-0.35) | 0.31 |
| ALT [μkat/l] | 0.49 (0.42-0.58) | | 0.43 (0.37-0.50) | 0.50 (0.42-0.60) | 0.79 | 0.29 (0.26-0.33) | 0.29 (0.26-0.33) | 0.34 (0.30-0.38) | 0.12 |
| fetuin-A [μg/ml] | 283 (261-305) | | 261 (241-281) | 247 (224-270) | 0.05 | 269 (250-287) | 274 (255-293) | 281 (262-301) | 0.37 |
|  |  | |  |  |  |  |  |  |  |
|  |  | |  |  |  |  |  |  |  |
|  |  | |  |  |  |  |  |  |  |
| 16:1n-9 |  | |  |  |  |  |  |  |  |
| FLI [Score points] | 38.3 (32.0-45.8) | | 37.4 (31.3-44.7) | 47.3 (39.5-56.6) | 0.08 | 13.6 (11.1-16.6) | 13.5 (11.1-16.4) | 12.2 (9.97-15.0) | 0.44 |
| GGT [μkat/l] | 0.62 (0.47-0.82) | | 0.49 (0.37-0.65) | 0.68 (0.51-0.90) | 0.54 | 0.24 (0.19-0.30) | 0.30 (0.24-0.37) | 0.19 (0.15-0.24) | 0.07 |
| ALT [μkat/l] | 0.43 (0.37-0.50) | | 0.49 (0.42-0.57) | 0.50 (0.43-0.58) | 0.19 | 0.31 (0.27-0.36) | 0.31 (0.27-0.35) | 0.30 (0.26-0.34) | 0.59 |
| fetuin-A [μg/ml] | 254 (234-274) | | 275 (255-295) | 261 (241-282) | 0.77 | 268 (249-287) | 284 (265-303) | 272 (252-291) | 0.99 |
|  |  | |  |  |  |  |  |  |  |
| 18:1n-7 |  | |  |  |  |  |  |  |  |
| FLI [Score points] | 39.6 (32.8-47.7) | | 40.9 (34.2-48.8) | 41.8 (34.4-50.7) | 0.71 | 14.4 (11.7-17.6) | 12.3 (10.1-15.0) | 12.7 (10.3-15.6) | 0.39 |
| GGT [μkat/l] | 0.52 (0.38-0.69) | | 0.60 (0.45-0.79) | 0.67 (0.49-0.90) | 0.26 | 0.29 (0.23-0.36) | 0.23 (0.19-0.29) | 0.21 (0.17-0.27) | 0.08 |
| ALT [μkat/l] | 0.45 (0.39-0.53) | | 0.47 (0.40-0.54) | 0.50 (0.42-0.59) | 0.42 | 0.31 (0.27-0.36) | 0.31 (0.28-0.36) | 0.29 (0.26-0.33) | 0.54 |
| fetuin-A [μg/ml] | 267 (246-287) | | 247 (228-267) | 277 (257-298) | 0.42 | 260 (240-280) | 279 (260-298) | 285 (265-305) | 0.10 |

^a^ In a multivariable linear regression analysis, we modeled the individual FA proportions as tertiles. The model was adjusted for age at recruitment, smoking status (never, past, current smoker <20 units/days, current smoker ≥20 units/days), alcohol intake (0, >0-6; >6-12; >12-24; >24-60; >60-96; >96 g/d), leisure time sports activity (no sports, ≤4 h/week, >4 h/week), biking (no biking, <2.5 h/week, 2.5-4.9 h/week, ≥5 h/week), hormone use in women (none, oral contraceptive, hormone replacement therapy [HRT]), education status (in or no training, vocational training, technical school, technical college or university degree), energy intake from the sum of mono- and disaccharides (%), energy intake from polysaccharides (%), energy intake from fat (%), BMI (kg/m^2^) and waist circumference (cm). We estimated geometric means and 95% confidence intervals (CI) in case of GGT, ALT and the FLI and arithmetic means and 95% CI in case of fetuin-A by FA tertiles and tested for statistical significance of linear trends across FA tertiles by modeling the median value of the FA within each tertile as a quantitative variable. *P* for trend value reflects whether the biomarker significantly increases or decreases across the FA tertiles.
