# Supplementary material for: Associations of Erythrocyte Fatty Acids in the De Novo Lipogenesis Pathway with Proxies of Liver Fat Accumulation in the EPIC-Potsdam Study
Source: PLoS One. 2015 May 18;10(5):e0127368. doi: 10.1371/journal.pone.0127368 (PMC4435749; doi:10.1371/journal.pone.0127368)
Supplement: S2 Table — Participants with a history of cancer, diabetes or cardiovascular diseases were excluded leaving 514 men and 877 women for this analysis. (DOCX) [file pone.0127368.s002.docx]

Table S2. Adjusted geometric means of the fatty liver index (FLI), plasma GGT and ALT and adjusted arithmetic means (95% CI) of plasma fetuin-A by tertiles of erythrocyte FA proportions, EPIC-Potsdam study ^a^. Participants with a history of cancer, diabetes and cardiovascular diseases were excluded leaving 514 men and 877 women for this analysis.

|  | | Men | | | | Women | | | |
| --- | --- | --- | --- | --- | --- | --- | --- | --- | --- |
|  | | Tertile of fatty acid | | | *p* for trend | Tertile of fatty acid | | | *p* for  trend |
|  | | 1 | 2 | 3 |  | 1 | 2 | 3 |  |
| 16:0 / 18:2n-6 (DNL-index) | | |  |  |  |  |  |  |  |
| FLI [Score points] | 37.2 (34.4-40.3) | | 38.6 (35.8-41.7) | 41.0 (37.9-44.4) | 0.10 | 11.6 (10.8-12.4) | 11.9 (11.1-12.8) | 11.8 (11.0-12.7) | 0.71 |
| GGT [μkat/l] | 0.42 (0.38-0.47) | | 0.49 (0.44-0.55) | 0.56 (0.50-0.62) | 0.0009 | 0.23 (0.21-0.25) | 0.23 (0.21-0.25) | 0.23 (0.21-0.25) | 0.93 |
| ALT [μkat/l] | 0.44 (0.41-0.47) | | 0.47 (0.44-0.50) | 0.50 (0.47-0.54) | 0.009 | 0.28 (0.27-0.30) | 0.29 (0.27-0.30) | 0.29 (0.27-0.30) | 0.73 |
| fetuin-A [μg/ml] | 252 (244-261) | | 259 (251-267) | 264 (256-273) | 0.07 | 264 (256-271) | 262 (255-270) | 271 (263-278) | 0.17 |
|  |  | |  |  |  |  |  |  |  |
| 14:0 |  | |  |  |  |  |  |  |  |
| FLI [Score points] | 36.8 (34.1-39.8) | | 36.7 (34.0-39.6) | 43.6 (40.4-47.1) | 0.002 | 11.5 (10.7-12.4) | 11.6 (10.8-12.4) | 12.3 (11.4-13.2) | 0.23 |
| GGT [μkat/l] | 0.46 (0.41-0.52) | | 0.45 (0.40-0.50) | 0.55 (0.49-0.62) | 0.02 | 0.23 (0.22-0.25) | 0.23 (0.21-0.24) | 0.24 (0.22-0.26) | 0.90 |
| ALT [μkat/l] | 0.45 (0.42-0.48) | | 0.47 (0.44-0.5) | 0.49 (0.45-0.52) | 0.09 | 0.29 (0.28-0.31) | 0.28 (0.27-0.30) | 0.28 (0.27-0.29) | 0.16 |
| fetuin-A [μg/ml] | 250 (242-258) | | 256 (247-264) | 270 (262-278) | 0.0008 | 255 (248-262) | 262 (255-269) | 280 (273-287) | <0.0001 |
|  |  | |  |  |  |  |  |  |  |
| 16:0 |  | |  |  |  |  |  |  |  |
| FLI [Score points] | 38.1 (35.2-41.2) | | 39.0 (36.1-42.1) | 39.7 (36.7-42.8) | 0.47 | 11.2 (10.5-12.1) | 12.1 (11.3-13.0) | 12.0 (11.1-12.8) | 0.24 |
| GGT [μkat/l] | 0.47 (0.42-0.53) | | 0.47 (0.42-0.52) | 0.52 (0.47-0.58) | 0.22 | 0.23 (0.21-0.25) | 0.24 (0.22-0.26) | 0.23 (0.21-0.25) | 0.71 |
| ALT [μkat/l] | 0.45 (0.42-0.49) | | 0.48 (0.44-0.51) | 0.47 (0.44-0.51) | 0.48 | 0.29 (0.28-0.31) | 0.29 (0.27-0.30) | 0.28 (0.26-0.29) | 0.07 |
| fetuin-A [μg/ml] | 248 (240-256) | | 257 (249-265) | 271 (263-279) | 0.0001 | 256 (249-263) | 261 (254-268) | 280 (273-287) | <0.0001 |
|  |  | |  |  |  |  |  |  |  |
| 16:1n-7 |  | |  |  |  |  |  |  |  |
| FLI [Score points] | 34.7 (32.1-37.4) | | 36.6 (34.0-39.5) | 46.5 (43.0-50.2) | <0.0001 | 10.4 (9.69-11.2) | 11.5 (10.8-12.4) | 13.6 (12.6-14.6) | <0.0001 |
| GGT [μkat/l] | 0.45 (0.40-0.50) | | 0.40 (0.36-0.45) | 0.64 (0.57-0.71) | <0.0001 | 0.22 (0.20-0.24) | 0.23 (0.21-0.25) | 0.25 (0.23-0.27) | 0.01 |
| ALT [μkat/l] | 0.45 (0.42-0.48) | | 0.43 (0.41-0.46) | 0.52 (0.49-0.56) | 0.002 | 0.28 (0.27-0.30) | 0.28 (0.27-0.29) | 0.30 (0.28-0.31) | 0.19 |
| fetuin-A [μg/ml] | 260 (252-268) | | 260 (252-269) | 255 (247-264) | 0.46 | 259 (252-267) | 265 (258-272) | 272 (265-279) | 0.02 |
|  |  | |  |  |  |  |  |  |  |
|  |  | |  |  |  |  |  |  |  |
| 16:1n-9 |  | |  |  |  |  |  |  |  |
| FLI [Score points] | 35.0 (32.4-37.8) | | 39.8 (36.9-43.0) | 42.3 (39.2-45.6) | 0.001 | 11.7 (10.9-12.5) | 11.8 (11.0-12.7) | 11.8 (11.0-12.7) | 0.88 |
| GGT [μkat/l] | 0.43 (0.39-0.48) | | 0.50 (0.45-0.56) | 0.53 (0.47-0.59) | 0.02 | 0.23 (0.21-0.25) | 0.23 (0.21-0.25) | 0.23 (0.21-0.25) | 0.88 |
| ALT [μkat/l] | 0.45 (0.42-0.49) | | 0.47 (0.44-0.50) | 0.48 (0.44-0.51) | 0.38 | 0.29 (0.28-0.30) | 0.30 (0.28-0.31) | 0.27 (0.26-0.29) | 0.04 |
| fetuin-A [μg/ml] | 250 (242-258) | | 266 (258-274) | 260 (252-268) | 0.20 | 254 (247-261) | 272 (265-279) | 271 (264-278) | 0.006 |
|  |  | |  |  |  |  |  |  |  |
| 18:1n-7 |  | |  |  |  |  |  |  |  |
| FLI [Score points] | 36.6 (33.9-39.6) | | 40.1 (37.2-43.3) | 40.1 (37.1-43.3) | 0.12 | 11.8 (11.0-12.7) | 11.6 (10.8-12.4) | 11.9 (11.1-12.8) | 0.87 |
| GGT [μkat/l] | 0.45 (0.41-0.51) | | 0.48 (0.43-0.53) | 0.53 (0.48-0.59) | 0.05 | 0.23 (0.21-0.25) | 0.22 (0.20-0.24) | 0.24 (0.22-0.26) | 0.35 |
| ALT [μkat/l] | 0.47 (0.43-0.50) | | 0.46 (0.43-0.49) | 0.48 (0.45-0.52) | 0.52 | 0.29 (0.27-0.30) | 0.27 (0.26-0.29) | 0.30 (0.28-0.31) | 0.26 |
| fetuin-A [μg/ml] | 260 (251-268) | | 252 (244-260) | 264 (256-272) | 0.44 | 264 (257-271) | 264 (257-271) | 269 (261-276) | 0.40 |

^a^ In a multivariable linear regression analysis, we modeled the individual FA proportions as tertiles. The model was adjusted for age at recruitment, smoking status (never, past, current smoker <20 units/days, current smoker ≥20 units/days), alcohol intake (0, >0-6; >6-12; >12-24; >24-60; >60-96; >96 g/d), leisure time sports activity (no sports, ≤4 h/week, >4 h/week), biking (no biking, <2.5 h/week, 2.5-4.9 h/week, ≥5 h/week), hormone use in women (none, oral contraceptive, hormone replacement therapy [HRT]), education status (in or no training, vocational training, technical school, technical college or university degree), energy intake from the sum of mono- and disaccharides (%), energy intake from polysaccharides (%), energy intake from fat (%), BMI (kg/m^2^) and waist circumference (cm). We estimated geometric means and 95% confidence intervals (CI) in case of GGT, ALT and the FLI and arithmetic means and 95% CI in case of fetuin-A by FA tertiles and tested for statistical significance of linear trends across FA tertiles by modeling the median value of the FA within each tertile as a quantitative variable. *P* for trend value reflects whether the biomarker significantly increases or decreases across the FA tertiles.
