# Supplementary material for: Associations of Erythrocyte Fatty Acids in the De Novo Lipogenesis Pathway with Proxies of Liver Fat Accumulation in the EPIC-Potsdam Study
Source: PLoS One. 2015 May 18;10(5):e0127368. doi: 10.1371/journal.pone.0127368 (PMC4435749; doi:10.1371/journal.pone.0127368)
Supplement: S3 Table — Men with a consumption of >30 g alcohol per day and women with a consumption of >20 g alcohol per day were excluded leaving 426 men and 883 women for this analysis. (DOCX) [file pone.0127368.s003.docx]

Table S3. Adjusted geometric means of the fatty liver index (FLI), plasma GGT and ALT and adjusted arithmetic means (95% CI) of plasma fetuin-A by tertiles of erythrocyte FA proportions, EPIC-Potsdam study^a^. Men with a consumption of >30 g alcohol per day and women with a consumption of >20 g alcohol per day were excluded leaving 426 men and 883 women for this analysis.

|  | | Men | | | | Women | | | |
| --- | --- | --- | --- | --- | --- | --- | --- | --- | --- |
|  | | Tertile of fatty acid | | | *p* for trend | Tertile of fatty acid | | | *p* for  trend |
|  | | 1 | 2 | 3 |  | 1 | 2 | 3 |  |
| 16:0 / 18:2n-6 (DNL-index) | | |  |  |  |  |  |  |  |
| FLI [Score points] | 35.3 (32.4-38.4) | | 37.9 (34.8-41.3) | 39.5 (36.2-43.1) | 0.08 | 12.3 (11.4-13.2) | 13.0 (12.1-13.9) | 12.7 (11.8-13.6) | 0.55 |
| GGT [μkat/l] | 0.38 (0.34-0.43) | | 0.43 (0.38-0.48) | 0.49 (0.43-0.55) | 0.004 | 0.23 (0.21-0.25) | 0.24 (0.22-0.25) | 0.24 (0.22-0.26) | 0.45 |
| ALT [μkat/l] | 0.43 (0.40-0.46) | | 0.45 (0.42-0.49) | 0.46 (0.43-0.50) | 0.18 | 0.29 (0.28-0.30) | 0.29 (0.28-0.30) | 0.29 (0.28-0.30) | 0.95 |
| fetuin-A [μg/ml] | 251 (242-260) | | 260 (251-270) | 266 (256-275) | 0.04 | 265 (258-272) | 262 (255-269) | 276 (268-283) | 0.04 |
|  |  | |  |  |  |  |  |  |  |
| 14:0 |  | |  |  |  |  |  |  |  |
| FLI [Score points] | 36.9 (33.9-40.1) | | 34.2 (31.4-37.2) | 42.0 (38.5-45.7) | 0.03 | 12.5 (11.6-13.4) | 12.2 (11.4-13.1) | 13.2 (12.3-14.2) | 0.27 |
| GGT [μkat/l] | 0.44 (0.39-0.49) | | 0.39 (0.35-0.44) | 0.46 (0.41-0.51) | 0.51 | 0.24 (0.22-0.26) | 0.23 (0.21-0.24) | 0.24 (0.22-0.26) | 0.88 |
| ALT [μkat/l] | 0.44 (0.41-0.47) | | 0.45 (0.42-0.48) | 0.45 (0.42-0.49) | 0.50 | 0.30 (0.29-0.31) | 0.29 (0.27-0.30) | 0.28 (0.27-0.30) | 0.09 |
| fetuin-A [μg/ml] | 254 (245-264) | | 253 (244-263) | 269 (260-279) | 0.02 | 257 (250-264) | 266 (259-273) | 280 (273-287) | <0.0001 |
|  |  | |  |  |  |  |  |  |  |
| 16:0 |  | |  |  |  |  |  |  |  |
| FLI [Score points] | 37.4 (34.3-40.7) | | 37.6 (34.5-40.9) | 37.6 (34.5-41.0) | 0.92 | 12.3 (11.4-13.2) | 12.7 (11.8-13.7) | 12.9 (12.0-13.9) | 0.39 |
| GGT [μkat/l] | 0.43 (0.38-0.48) | | 0.41 (0.37-0.46) | 0.44 (0.40-0.50) | 0.68 | 0.23 (0.22-0.25) | 0.23 (0.21-0.25) | 0.24 (0.22-0.26) | 0.44 |
| ALT [μkat/l] | 0.45 (0.42-0.48) | | 0.45 (0.42-0.48) | 0.44 (0.41-0.48) | 0.83 | 0.30 (0.29-0.31) | 0.29 (0.27-0.30) | 0.28 (0.27-0.30) | 0.11 |
| fetuin-A [μg/ml] | 252 (243-261) | | 253 (244-262) | 272 (263-281) | 0.003 | 258 (250-265) | 263 (256-270) | 283 (276-290) | <0.0001 |
|  |  | |  |  |  |  |  |  |  |
| 16:1n-7 |  | |  |  |  |  |  |  |  |
| FLI [Score points] | 34.0 (31.2-37.0) | | 36.1 (33.2-39.3) | 43.0 (39.5-46.8) | 0.0001 | 11.2 (10.4-12.0) | 12.6 (11.7-13.5) | 14.3 (13.3-15.4) | <0.0001 |
| GGT [μkat/l] | 0.42 (0.37-0.47) | | 0.37 (0.33-0.41) | 0.51 (0.46-0.57) | 0.01 | 0.22 (0.20-0.24) | 0.23 (0.22-0.25) | 0.26 (0.24-0.28) | 0.01 |
| ALT [μkat/l] | 0.45 (0.42-0.48) | | 0.42 (0.39-0.45) | 0.47 (0.44-0.51) | 0.30 | 0.29 (0.28-0.30) | 0.28 (0.27-0.30) | 0.30 (0.28-0.31) | 0.36 |
| fetuin-A [μg/ml] | 266 (256-275) | | 256 (247-265) | 255 (245-264) | 0.13 | 261 (254-269) | 269 (262-276) | 273 (266-280) | 0.04 |
|  |  | |  |  |  |  |  |  |  |
|  |  | |  |  |  |  |  |  |  |
| 16:1n-9 |  | |  |  |  |  |  |  |  |
| FLI [Score points] | 35.2 (32.3-38.3) | | 36.5 (33.5-39.7) | 41.2 (37.9-44.9) | 0.01 | 12.8 (11.9-13.7) | 12.7 (11.8-13.6) | 12.5 (11.6-13.4) | 0.65 |
| GGT [μkat/l] | 0.42 (0.37-0.47) | | 0.40 (0.36-0.45) | 0.47 (0.41-0.52) | 0.17 | 0.23 (0.22-0.25) | 0.24 (0.22-0.26) | 0.24 (0.22-0.25) | 0.97 |
| ALT [μkat/l] | 0.44 (0.41-0.47) | | 0.45 (0.42-0.48) | 0.46 (0.42-0.49) | 0.47 | 0.29 (0.28-0.31) | 0.29 (0.28-0.31) | 0.28 (0.27-0.29) | 0.11 |
| fetuin-A [μg/ml] | 253 (244-262) | | 265 (255-274) | 259 (250-269) | 0.50 | 257 (250-264) | 276 (269-283) | 270 (263-277) | 0.05 |
|  |  | |  |  |  |  |  |  |  |
| 18:1n-7 |  | |  |  |  |  |  |  |  |
| FLI [Score points] | 36.0 (33.0-39.3) | | 38.7 (35.6-42.2) | 37.9 (34.8-41.3) | 0.43 | 12.8 (11.9-13.7) | 12.4 (11.5-13.3) | 12.8 (11.9-13.8) | 0.97 |
| GGT [μkat/l] | 0.41 (0.36-0.46) | | 0.43 (0.38-0.48) | 0.45 (0.40-0.51) | 0.21 | 0.23 (0.21-0.25) | 0.23 (0.21-0.25) | 0.25 (0.23-0.27) | 0.28 |
| ALT [μkat/l] | 0.45 (0.42-0.48) | | 0.44 (0.41-0.47) | 0.45 (0.42-0.48) | 1.00 | 0.29 (0.28-0.31) | 0.28 (0.27-0.29) | 0.30 (0.28-0.31) | 0.51 |
| fetuin-A [μg/ml] | 264 (255-274) | | 253 (243-262) | 260 (250-269) | 0.50 | 264 (257-272) | 265 (258-272) | 274 (267-281) | 0.07 |

^a^ In a multivariable linear regression analysis, we modeled the individual FA proportions as tertiles. The model was adjusted for age at recruitment, smoking status (never, past, current smoker <20 units/days, current smoker ≥20 units/days), alcohol intake (0, >0-6; >6-12; >12-24; >24-60; >60-96; >96 g/d), leisure time sports activity (no sports, ≤4 h/week, >4 h/week), biking (no biking, <2.5 h/week, 2.5-4.9 h/week, ≥5 h/week), hormone use in women (none, oral contraceptive, hormone replacement therapy [HRT]), education status (in or no training, vocational training, technical school, technical college or university degree), energy intake from the sum of mono- and disaccharides (%), energy intake from polysaccharides (%), energy intake from fat (%), BMI (kg/m^2^) and waist circumference (cm). We estimated geometric means and 95% confidence intervals (CI) in case of GGT, ALT and the FLI and arithmetic means and 95% CI in case of fetuin-A by FA tertiles and tested for statistical significance of linear trends across FA tertiles by modeling the median value of the FA within each tertile as a quantitative variable. *P* for trend value reflects whether the biomarker significantly increases or decreases across the FA tertiles.
