# Supplementary material for: Associations of Erythrocyte Fatty Acids in the De Novo Lipogenesis Pathway with Proxies of Liver Fat Accumulation in the EPIC-Potsdam Study
Source: PLoS One. 2015 May 18;10(5):e0127368. doi: 10.1371/journal.pone.0127368 (PMC4435749; doi:10.1371/journal.pone.0127368)
Supplement: S4 Table — Participants who were former heavy drinkers or consumed alcohol occasionally heavy or always heavy during their lifetime were excluded leaving 463 men and 920 women for this analysis. (DOCX) [file pone.0127368.s004.docx]

Table S4. Adjusted geometric means of the fatty liver index (FLI), plasma GGT and ALT and adjusted arithmetic means (95% CI) of plasma fetuin-A by tertiles of erythrocyte FA proportions, EPIC-Potsdam study^a^. Participants who were former heavy drinkers or consumed alcohol occasionally heavy or always heavy during their lifetime were excluded, leaving 463 men and 920 women for this analysis.

|  | | Men | | | | Women | | | |
| --- | --- | --- | --- | --- | --- | --- | --- | --- | --- |
|  | | Tertile of fatty acid | | | *p* for trend | Tertile of fatty acid | | | *p* for  trend |
|  | | 1 | 2 | 3 |  | 1 | 2 | 3 |  |
| 16:0 / 18:2n-6 (DNL-index) | | |  |  |  |  |  |  |  |
| FLI [Score points] | 37.5 (34.6-40.6) | | 40.1 (37.1-43.3) | 39.5 (36.4-42.7) | 0.40 | 12.1 (11.3-13) | 12.5 (11.7-13.5) | 12.5 (11.6-13.4) | 0.55 |
| GGT [μkat/l] | 0.43 (0.38-0.48) | | 0.48 (0.43-0.54) | 0.49 (0.44-0.54) | 0.13 | 0.23 (0.21-0.25) | 0.24 (0.22-0.26) | 0.24 (0.22-0.25) | 0.73 |
| ALT [μkat/l] | 0.44 (0.41-0.47) | | 0.48 (0.45-0.51) | 0.44 (0.41-0.47) | 0.81 | 0.29 (0.27-0.30) | 0.29 (0.27-0.30) | 0.29 (0.28-0.30) | 0.83 |
| fetuin-A [μg/ml] | 250 (241-259) | | 261 (252-270) | 259 (250-268) | 0.17 | 264 (257-271) | 263 (256-270) | 275 (268-282) | 0.03 |
|  |  | |  |  |  |  |  |  |  |
| 14:0 |  | |  |  |  |  |  |  |  |
| FLI [Score points] | 37.9 (35.0-41.0) | | 36.4 (33.7-39.3) | 43.0 (39.7-46.4) | 0.02 | 12.1 (11.3-13.0) | 12.2 (11.3-13.0) | 12.8 (12.0-13.8) | 0.26 |
| GGT [μkat/l] | 0.48 (0.43-0.54) | | 0.44 (0.39-0.49) | 0.47 (0.42-0.53) | 0.85 | 0.24 (0.22-0.26) | 0.23 (0.21-0.25) | 0.24 (0.22-0.25) | 0.75 |
| ALT [μkat/l] | 0.45 (0.42-0.48) | | 0.46 (0.43-0.49) | 0.45 (0.42-0.49) | 0.91 | 0.29 (0.28-0.31) | 0.29 (0.27-0.30) | 0.28 (0.27-0.30) | 0.29 |
| fetuin-A [μg/ml] | 253 (244-262) | | 252 (244-261) | 265 (256-274) | 0.05 | 256 (249-263) | 266 (259-273) | 279 (273-286) | <0.0001 |
|  |  | |  |  |  |  |  |  |  |
| 16:0 |  | |  |  |  |  |  |  |  |
| FLI [Score points] | 38.6 (35.6-41.7) | | 39.0 (36.0-42.2) | 39.4 (36.4-42.6) | 0.70 | 12.0 (11.2-12.9) | 12.6 (11.7-13.5) | 12.5 (11.6-13.4) | 0.53 |
| GGT [μkat/l] | 0.46 (0.42-0.52) | | 0.46 (0.41-0.51) | 0.47 (0.42-0.52) | 0.87 | 0.23 (0.21-0.25) | 0.24 (0.22-0.25) | 0.24 (0.22-0.26) | 0.62 |
| ALT [μkat/l] | 0.45 (0.42-0.49) | | 0.45 (0.42-0.49) | 0.46 (0.42-0.49) | 0.87 | 0.30 (0.28-0.31) | 0.29 (0.27-0.30) | 0.28 (0.27-0.29) | 0.13 |
| fetuin-A [μg/ml] | 249 (240-258) | | 252 (244-261) | 269 (260-277) | 0.002 | 257 (250-264) | 263 (256-270) | 282 (275-289) | <0.0001 |
|  |  | |  |  |  |  |  |  |  |
| 16:1n-7 |  | |  |  |  |  |  |  |  |
| FLI [Score points] | 35.4 (32.7-38.3) | | 37.9 (35.1-41.0) | 44.2 (40.8-47.8) | 0.0001 | 10.9 (10.2-11.7) | 12.4 (11.5-13.3) | 14.0 (13.0-15.0) | <0.0001 |
| GGT [μkat/l] | 0.45 (0.40-0.50) | | 0.39 (0.35-0.44) | 0.56 (0.51-0.63) | 0.004 | 0.22 (0.20-0.24) | 0.23 (0.22-0.25) | 0.25 (0.23-0.27) | 0.01 |
| ALT [μkat/l] | 0.45 (0.42-0.49) | | 0.43 (0.40-0.46) | 0.48 (0.45-0.52) | 0.21 | 0.28 (0.27-0.30) | 0.28 (0.27-0.30) | 0.30 (0.28-0.31) | 0.21 |
| fetuin-A [μg/ml] | 260 (251-269) | | 258 (249-267) | 252 (243-261) | 0.20 | 262 (255-269) | 268 (262-275) | 272 (264-279) | 0.07 |
|  |  | |  |  |  |  |  |  |  |
|  |  | |  |  |  |  |  |  |  |
| 16:1n-9 |  | |  |  |  |  |  |  |  |
| FLI [Score points] | 36.9 (34.1-39.9) | | 39.4 (36.4-42.6) | 40.8 (37.7-44.1) | 0.09 | 12.4 (11.5-13.3) | 12.5 (11.6-13.4) | 12.3 (11.5-13.2) | 0.87 |
| GGT [μkat/l] | 0.45 (0.41-0.50) | | 0.47 (0.42-0.52) | 0.48 (0.43-0.53) | 0.53 | 0.23 (0.21-0.25) | 0.24 (0.22-0.26) | 0.23 (0.22-0.25) | 0.96 |
| ALT [μkat/l] | 0.44 (0.41-0.47) | | 0.46 (0.42-0.49) | 0.46 (0.43-0.50) | 0.35 | 0.29 (0.28-0.30) | 0.29 (0.28-0.31) | 0.28 (0.27-0.29) | 0.12 |
| fetuin-A [μg/ml] | 249 (241-258) | | 260 (252-269) | 260 (252-269) | 0.11 | 257 (250-264) | 274 (267-281) | 271 (264-277) | 0.04 |
|  |  | |  |  |  |  |  |  |  |
| 18:1n-7 |  | |  |  |  |  |  |  |  |
| FLI [Score points] | 37.4 (34.6-40.5) | | 41.1 (38.0-44.4) | 38.6 (35.6-41.7) | 0.63 | 12.4 (11.6-13.4) | 12.1 (11.3-13.0) | 12.5 (11.7-13.5) | 0.88 |
| GGT [μkat/l] | 0.44 (0.39-0.49) | | 0.48 (0.43-0.54) | 0.47 (0.42-0.53) | 0.34 | 0.23 (0.22-0.25) | 0.23 (0.21-0.24) | 0.24 (0.23-0.26) | 0.35 |
| ALT [μkat/l] | 0.45 (0.42-0.49) | | 0.45 (0.42-0.48) | 0.46 (0.42-0.49) | 0.94 | 0.29 (0.28-0.31) | 0.28 (0.27-0.29) | 0.29 (0.28-0.30) | 0.91 |
| fetuin-A [μg/ml] | 258 (249-267) | | 253 (245-262) | 259 (250-267) | 0.92 | 265 (258-272) | 264 (258-271) | 272 (265-279) | 0.16 |

^a^ In a multivariable linear regression analysis, we modeled the individual FA proportions as tertiles. The model was adjusted for age at recruitment, smoking status (never, past, current smoker <20 units/days, current smoker ≥20 units/days), alcohol intake (0, >0-6; >6-12; >12-24; >24-60; >60-96; >96 g/d), leisure time sports activity (no sports, ≤4 h/week, >4 h/week), biking (no biking, <2.5 h/week, 2.5-4.9 h/week, ≥5 h/week), hormone use in women (none, oral contraceptive, hormone replacement therapy [HRT]), education status (in or no training, vocational training, technical school, technical college or university degree), energy intake from the sum of mono- and disaccharides (%), energy intake from polysaccharides (%), energy intake from fat (%), BMI (kg/m^2^) and waist circumference (cm). We estimated geometric means and 95% confidence intervals (CI) in case of GGT, ALT and the FLI and arithmetic means and 95% CI in case of fetuin-A by FA tertiles and tested for statistical significance of linear trends across FA tertiles by modeling the median value of the FA within each tertile as a quantitative variable. *P* for trend value reflects whether the biomarker significantly increases or decreases across the FA tertiles.
