# Supplementary material for: Associations of Erythrocyte Fatty Acids in the De Novo Lipogenesis Pathway with Proxies of Liver Fat Accumulation in the EPIC-Potsdam Study
Source: PLoS One. 2015 May 18;10(5):e0127368. doi: 10.1371/journal.pone.0127368 (PMC4435749; doi:10.1371/journal.pone.0127368)
Supplement: S5 Table — (DOCX) [file pone.0127368.s005.docx]

Table S5. Adjusted geometric means of the fatty liver index (FLI), plasma GGT and ALT and adjusted arithmetic means (95% CI) of plasma fetuin-A by tertiles of erythrocyte FA proportions, EPIC-Potsdam study^a^. Participants with HbA1c values ≥5.7 % were excluded, leaving 430 men and 785 women for this analysis.

|  | | Men | | | | Women | | | |
| --- | --- | --- | --- | --- | --- | --- | --- | --- | --- |
|  | | Tertile of fatty acid | | | *p* for trend | Tertile of fatty acid | | | *p* for  trend |
|  | | 1 | 2 | 3 |  | 1 | 2 | 3 |  |
| 16:0 / 18:2n-6 (DNL-index) | | |  |  |  |  |  |  |  |
| FLI [Score points] | 35.8 (32.8-39.0) | | 38.1 (35.0-41.4) | 40.3 (37.0-43.9) | 0.06 | 10.6 (9.79-11.4) | 11.3 (10.5-12.2) | 10.9 (10.1-11.8) | 0.57 |
| GGT [μkat/l] | 0.41 (0.36-0.47) | | 0.47 (0.42-0.53) | 0.56 (0.49-0.63) | 0.001 | 0.22 (0.20-0.23) | 0.23 (0.21-0.25) | 0.22 (0.20-0.24) | 0.91 |
| ALT [μkat/l] | 0.43 (0.40-0.47) | | 0.46 (0.42-0.49) | 0.49 (0.45-0.53) | 0.02 | 0.28 (0.27-0.30) | 0.28 (0.27-0.30) | 0.28 (0.27-0.29) | 0.88 |
| fetuin-A [μg/ml] | 254 (244-263) | | 259 (249-268) | 261 (251-270) | 0.36 | 260 (253-268) | 259 (252-267) | 271 (263-278) | 0.06 |
|  |  | |  |  |  |  |  |  |  |
| 14:0 |  | |  |  |  |  |  |  |  |
| FLI [Score points] | 36.2 (33.3-39.3) | | 35.7 (32.9-38.8) | 42.5 (39.1-46.2) | 0.006 | 11.1 (10.3-12.0) | 10.4 (9.65-11.2) | 11.3 (10.5-12.1) | 0.77 |
| GGT [μkat/l] | 0.45 (0.40-0.51) | | 0.44 (0.39-0.50) | 0.54 (0.48-0.61) | 0.03 | 0.24 (0.22-0.26) | 0.21 (0.20-0.23) | 0.22 (0.20-0.24) | 0.16 |
| ALT [μkat/l] | 0.43 (0.40-0.47) | | 0.47 (0.43-0.50) | 0.48 (0.44-0.51) | 0.09 | 0.29 (0.28-0.31) | 0.28 (0.27-0.29) | 0.27 (0.26-0.29) | 0.04 |
| fetuin-A [μg/ml] | 249 (240-258) | | 258 (249-268) | 266 (257-275) | 0.01 | 257 (249-264) | 262 (254-269) | 272 (265-280) | 0.004 |
|  |  | |  |  |  |  |  |  |  |
| 16:0 |  | |  |  |  |  |  |  |  |
| FLI [Score points] | 36.5 (33.6-39.7) | | 39.3 (36.1-42.7) | 38.3 (35.2-41.6) | 0.44 | 10.8 (10.0-11.7) | 10.6 (9.85-11.4) | 11.4 (10.5-12.3) | 0.34 |
| GGT [μkat/l] | 0.44 (0.39-0.50) | | 0.51 (0.45-0.58) | 0.48 (0.43-0.54) | 0.33 | 0.23 (0.21-0.25) | 0.22 (0.20-0.23) | 0.22 (0.21-0.24) | 0.76 |
| ALT [μkat/l] | 0.44 (0.41-0.48) | | 0.49 (0.45-0.52) | 0.45 (0.41-0.48) | 0.97 | 0.29 (0.28-0.31) | 0.28 (0.27-0.30) | 0.27 (0.26-0.28) | 0.03 |
| fetuin-A [μg/ml] | 249 (240-258) | | 256 (247-265) | 268 (259-278) | 0.004 | 257 (250-265) | 258 (250-265) | 276 (268-283) | 0.0005 |
|  |  | |  |  |  |  |  |  |  |
| 16:1n-7 |  | |  |  |  |  |  |  |  |
| FLI [Score points] | 33.8 (31.1-36.7) | | 36 (33.2-39.1) | 45.1 (41.5-49.1) | <0.0001 | 9.83 (9.13-10.6) | 10.6 (9.84-11.4) | 12.5 (11.6-13.5) | <0.0001 |
| GGT [μkat/l] | 0.43 (0.38-0.48) | | 0.39 (0.35-0.44) | 0.64 (0.57-0.73) | <0.0001 | 0.21 (0.19-0.22) | 0.23 (0.21-0.25) | 0.24 (0.22-0.26) | 0.03 |
| ALT [μkat/l] | 0.45 (0.42-0.48) | | 0.42 (0.39-0.45) | 0.52 (0.48-0.56) | 0.008 | 0.28 (0.27-0.30) | 0.27 (0.26-0.29) | 0.29 (0.27-0.30) | 0.52 |
| fetuin-A [μg/ml] | 263 (254-273) | | 257 (248-266) | 253 (244-263) | 0.17 | 258 (250-266) | 264 (257-272) | 268 (261-276) | 0.07 |
|  |  | |  |  |  |  |  |  |  |
|  |  | |  |  |  |  |  |  |  |
| 16:1n-9 |  | |  |  |  |  |  |  |  |
| FLI [Score points] | 35.5 (32.7-38.6) | | 37.9 (34.9-41.2) | 40.7 (37.5-44.3) | 0.03 | 11.0 (10.2-11.8) | 10.9 (10.1-11.8) | 10.9 (10.1-11.7) | 0.86 |
| GGT [μkat/l] | 0.44 (0.39-0.50) | | 0.47 (0.42-0.53) | 0.52 (0.46-0.59) | 0.07 | 0.22 (0.20-0.24) | 0.22 (0.21-0.24) | 0.22 (0.20-0.24) | 0.99 |
| ALT [μkat/l] | 0.45 (0.41-0.48) | | 0.46 (0.43-0.50) | 0.47 (0.43-0.50) | 0.40 | 0.29 (0.27-0.30) | 0.29 (0.27-0.30) | 0.27 (0.26-0.28) | 0.04 |
| fetuin-A [μg/ml] | 250 (241-259) | | 265 (255-274) | 259 (249-268) | 0.32 | 256 (248-263) | 265 (258-272) | 270 (263-277) | 0.01 |
|  |  | |  |  |  |  |  |  |  |
| 18:1n-7 |  | |  |  |  |  |  |  |  |
| FLI [Score points] | 34.5 (31.7-37.5) | | 39.8 (36.7-43.3) | 39.9 (36.7-43.4) | 0.02 | 10.9 (10.1-11.8) | 10.8 (10.1-11.7) | 11.0 (10.2-11.9) | 0.83 |
| GGT [μkat/l] | 0.43 (0.38-0.48) | | 0.47 (0.41-0.53) | 0.55 (0.48-0.62) | 0.005 | 0.22 (0.20-0.24) | 0.22 (0.20-0.23) | 0.23 (0.21-0.25) | 0.26 |
| ALT [μkat/l] | 0.44 (0.41-0.48) | | 0.45 (0.42-0.49) | 0.48 (0.45-0.52) | 0.16 | 0.28 (0.27-0.30) | 0.27 (0.26-0.28) | 0.29 (0.28-0.31) | 0.27 |
| fetuin-A [μg/ml] | 256 (247-265) | | 257 (248-266) | 260 (251-270) | 0.54 | 260 (252-268) | 263 (255-270) | 268 (260-276) | 0.18 |

^a^ In a multivariable linear regression analysis, we modeled the individual FA proportions as tertiles. The model was adjusted for age at recruitment, smoking status (never, past, current smoker <20 units/days, current smoker ≥20 units/days), alcohol intake (0, >0-6; >6-12; >12-24; >24-60; >60-96; >96 g/d), leisure time sports activity (no sports, ≤4 h/week, >4 h/week), biking (no biking, <2.5 h/week, 2.5-4.9 h/week, ≥5 h/week), hormone use in women (none, oral contraceptive, hormone replacement therapy [HRT]), education status (in or no training, vocational training, technical school, technical college or university degree), energy intake from the sum of mono- and disaccharides (%), energy intake from polysaccharides (%), energy intake from fat (%), BMI (kg/m^2^) and waist circumference (cm). We estimated geometric means and 95% confidence intervals (CI) in case of GGT, ALT and the FLI and arithmetic means and 95% CI in case of fetuin-A by FA tertiles and tested for statistical significance of linear trends across FA tertiles by modeling the median value of the FA within each tertile as a quantitative variable. *P* for trend value reflects whether the biomarker significantly increases or decreases across the FA tertiles.
